# ﻿A new species of *Myotis* from China with notes on the *siligorensis* species group (Chiroptera, Vespertilionidae)

**DOI:** 10.3897/zookeys.1258.145290

**Published:** 2025-11-07

**Authors:** Mikhail Petrovich Tiunov, Sen Liu, Jiang Feng, Pipat Soisook, Tinglei Jiang

**Affiliations:** 1 Federal Science Center of East Asian Terrestrial Biodiversity, Far East Branch, Russian Academy of Sciences, Pr-t 100-let Vladivostoka 159, Vladivostok 690022, Russia Federal Science Center of East Asian Terrestrial Biodiversity, Russian Academy of Sciences Vladivostok Russia; 2 College of Life Sciences, Henan Normal University, 46 Jianshe East Road, Xinxiang 453007, China Henan Normal University Xinxiang China; 3 Jilin Provincial Key Laboratory of Animal Resource Conservation and Utilization, Northeast Normal University, Changchun 130117, China Northeast Normal University Changchun China; 4 Princess Maha Chakri Sirindhorn Natural History Museum, Prince of Songkla University, Hat Yai, Songkhla 90112, Thailand Prince of Songkla University Songkhla Thailand

**Keywords:** Baculum, bats, morphology, new species, taxonomy, tragus

## Abstract

On the basis of molecular and morphological studies of samples collected in China, a new *Myotis* species belonging to the *siligorensis* group is described, *Myotis kalkoae* Tiunov, Jiang, & Liu, **sp. nov.** The species rank of *M. sowerbyi* and *M. alticraniatus* was confirmed. All three taxa under consideration belong to different genetic lines and can be distinguished from each other and from other morphologically similar species based on the shape of their baculum and tragus. The difficulties of taxonomy within the *M. siligorensis* species group are discussed.

## ﻿Introduction

*Myotis* is one of the most diverse chiropteran genera (Koopman 1994; Simmons 2005). The content, worldwide distribution, and separate taxonomic position (as a member of a separate subfamily; refer to Hoofer and van den Busche 2003) of this genus highlight the importance of taxonomic studies within this group. Molecular genetics methods have been applied for the taxonomic studies of *Myotis* since the early 2000s (Ruedi and Mayer 2001), resulting in several rearrangements at the taxonomic levels (e.g., Stadelmann et al. 2004, 2007; Lack et al. 2010; Larsen et al. 2012; Novaes et al. 2021; Saikia et al. 2025), which contributed to increased interest in taxonomic studies of bats in general. However, some of the recent successful taxonomic publications (not particularly concerning *Myotis*) are not based on molecular data (Csorba et al. 2011; Reeder et al. 2013).

**Figure 1.**
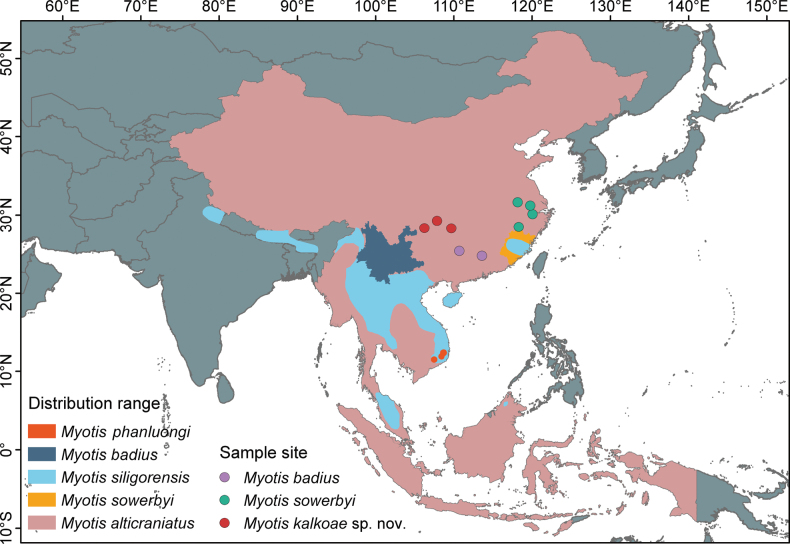
Distribution map of the forms within the *M. siligorensis* species group and the sampling locations of the three species, namely, *M. badius*, *M. sowerbyi*, and *M. kalkoae* sp. nov., used in this study. The ranges for *M. badius* and *M. siligorensis* are based on the IUCN Red List of Threatened Species (https://www.iucnredlist.org/), whereas the ranges for *M. phanluongi*, *M. sowerbyi*, and *M. alticraniatus* are based on the Mammal Diversity Database (2005) (https://www.mammaldiversity.org/). Note that the ranges of these species are currently controversial.

Some species groups of tropical *Myotis* have been studied insufficiently, and their taxonomic status and species delimitation require further investigations. Previous studies have revealed cryptic diversity within the *Myotis siligorensis* species group, and two new species were described, namely, *M. phanluongi* Borisenko, Kruskop & Ivanova, 2008 and *M. badius* Tiunov, Kruskop & Feng, 2011. However, the taxonomy of this group remains unclear to date, although new undescribed forms have been identified (Francis et al. 2010; Ruedi et al. 2013). For example, two subspecies of *M. siligorensis* (*alticraniatus* and *sowerbyi*) have been elevated to the species level, and *M. alticraniatus* Osgood, 1932 may include three subspecies (*alticraniatus*, *thaianus*, and *badius*) (Ruedi et al. 2015, 2021). However, the status of the subspecies of *M. alticraniatus* is debatable, especially for *M. alticraniatus badius*. Therefore, *M. badius* is considered a separate species and here raised to specific status (Fig. 1).

Kawai et al. (2003) published the molecular taxonomic data on the rare bat species *Myotis davidii* (Peters, 1869), a species endemic to East Asia. Since these data were placed in GenBank, the number of fresh records of this species in China has increased markedly. Further study was conducted on the genetic diversity of 126 individuals, which were originally identified as *Myotis davidii*, obtained from seventeen Chinese localities (You et al. 2010). This study, which was based on nuclear and mitochondrial DNA markers, demonstrated that individuals can be subdivided into three groups based on their geographical origin. Moreover, P. Benda investigated the holotype of *Vespertilio davidii* (Peters, 1869) and allocated it into a synonymy of the ‘*mystacinus*’ species group, considering that “this name represents the senior synonym of the whole group of morphotypes included … into species rank of *Myotis nipalensis*...” (Benda and Karatas 2005). Later, P. Benda accepted *M. davidii* as a valid and senior name for the *M. nipalensis / aurascens* complex (Benda et al. 2012), placing it apart from the *siligorensis* species group. Ruedi et al. (2021) also considered *M. davidii* to be a well-defined species but distinguished it from *M. nipalensis*, which inhabits the southern slopes of the Himalayan region, as a separate species.

S. Kruskop also examined three specimens of *M. davidii* from Central China (including the holotype MNHN1987-296), and confirmed (pers. comm., oral) that all three correspond to the *M. mystacinus* species group in skull shape and tooth structure (although they represent the extreme variants of the P3 displacement). According to their morphological characteristics, none of the samples (identified genetically as *Myotis davidii* sensu Kawai et al. 2003) fit the original description of this species. According to Tate (1941), “A specimen, U.S.N.M. 219175 from Chi-li, has a forearm length of 33 mm, thumbs and wing attachment as in true *daubentonii*, the skull with full braincase, large anteorbital foramen of *Leuconoe*, but with the muzzle and palate so much shortened that p^3^ is wholly excluded and contact is established between p^4^ and p^2^.” All the individuals that were identified previously as *M. davidii* based on the molecular analysis (You et al. 2010) exhibited a different dental structure. A single specimen in the collection of the Museum of Natural History (London), originally labeled *M. davidii*NHMUK 9.1.1.1, does not present skull and teeth proportions typical of the *M. siligorensis* species group, whereas Kawai’s ‘*M. davidii*’ are definitely within the *siligorensis* group (clade IX sensu Ruedi et al. 2013; refer also to Zhang et al. 2009).

It was assumed that all the questionable Chinese samples studied belonged to the *Myotis siligorensis* species group, although their affiliations with a particular species remained uncertain. All of these specimens possess lower molars of the nyctalodont or seminyctalodont type, with a postcristid connected chiefly to a hypoconulid (Menu 1987), a feature that is typical of the *M. siligorensis* group (although not for all its members), in contrast to the vast majority of the other myotines, which are myotodonts. The individuals also possess skulls of recognizable shape, with a high and rounded braincase and a low rostral portion, which divides them from other nyctalodont myotines, such as *Submyotodon latirostris* and *S. caliginosus* (Ruedi et al. 2015).

Most species in the *M. siligorensis* species group are quite similar in overall size and skull proportions. However, our study shows that they differ in the shapes of their tragus and penial bone (baculum). Despite a certain level of individual variation, the latter structure is known to be a useful species diagnostic feature, at least in some Vespertilionid genera, separating even the morphologically similar species (e.g., Thomas 1915; Topal 1958; Brown 1967; Hill and Harrison 1987; Benda and Tsytsulina 2000; Matveev et al. 2005). Based on these findings, the bacular morphologies of all taxa belonging to the group *M. siligorensis* and the material belonging to the different genetic lineages previously assigned to ‘*M. davidii*’ were studied. Thus, using an integrative combination of molecular and morphological analyses, a new species was identified in the *M. siligorensis* group, *Myotis kalkoae* Tiunov, Jiang, & Liu, sp. nov.

## ﻿Materials and methods

### ﻿Morphological and morphometric studies

Three specimens of the *Myotis kalkoae* sp. nov. were captured during the MPT’s field work and fixed in 75% ethanol, and eighty-four samples were used for qualitative and quantitative morphological comparisons (adult individuals of both sexes; dry- or alcohol-preserved skins with extracted skulls; see Suppl. material 1) (Fig. 1). The abbreviations of the collections are as follows:

**FMNH** Field Museum of Natural History, Chicago, USA

**HNHM** Hungarian Natural History Museum, Budapest, Hungary

**HZM** Harrison Institute, formerly the Harrison Zoological Museum, Sevenoaks, Kent, Great Britain

**IBSS** Institute of Biology and Soil Science, Far East Branch of the Russian Academy of Sciences, Vladivostok, Russia

**MNH** Museum of Natural History, London, Great Britain

**MNHN** National Museum of Natural History, Paris, France

**NHMUK** Museum of Natural History, London, Great Britain

**NNU** Northeast Normal University, Changchun, China

**ROM** Royal Ontario Museum, Toronto, Canada

**SMF** Senckenberg Museum of Natural History, Frankfurt am Main, Germany

**ZMMU** Zoological Museum of Moscow State University, Moscow, Russia

External measurements were taken to the nearest 0.1 mm using a dial caliper. In the laboratory, a set of 19 cranial measurements was taken to the nearest 0.01 mm using an electronic caliper in combination with a binocular microscope.

The following external measurements were taken:

**HB** head and body length,

**T** tail length,

**TT** length of the free tail tip,

**E** ear length,

**Tr** tragus length,

**Tib** tibia length,

**F** foot length (including claws, measured to the most remote part of the claw),

**FA** forearm length,

**Mc** length of the first digit, including the claw, length of the metacarpal of the second digit, and lengths of the metacarpals,

**Ph** phalanxes of the third through fifth digits.

All measurements for the wings were taken using the right wing. The following cranial characteristics were measured (appropriate abbreviations are provided in parentheses):

**CBL** condylobasal length,

**CCL** condylocanine length,

**W** width of the skull at the level of the auditory bullae,

**BCW** width of the braincase,

**BCH** height of the braincase posterior to the auditory bullae,

**IOW** least interorbital width,

**ZW** zygomatic width,

**WR** rostral width at the level of the preorbital foramina,

**LR** rostral length from the preorbital foramen to the anterior edges of alveolus of the inner incisor,

**CM3** C–M3 crown length,

‘**pseudodiastema’ PD** length of the interval between the cingulum of the upper canine and large premolar,

**P4**M3 molariform tooth row length,

**CC** crown width between the outer margins of the upper canines,

**M3M3** crown width between the outer margins of M3,

**lmd** lower jaw length from the alveolus of I 1 to the articulated process,

**hmd** lower jaw height from the level of the tip of the coronoid process,

**MCM3** crown length of the maxillary tooth row.

This list of measurements was limited to 16 items for statistical purposes. Only intact skulls with a complete set of measurements were used in the analysis. In order to assess the pattern of variation in the quantitative characteristics, principal component (PC) analysis and discriminant factor (DF) analysis were performed for cranial measurements using the appropriate modules of STATISTICA for Windows (Stat-Soft Inc. 1999). In the PC analysis, the measurements were standardized [(raw score - mean)/SD] to decrease the influence of the overall size.

The shapes of the tragus and baculum were examined in three samples of *M. kalkoae* sp. nov., 11 of *M. badius*, nine of *M. sowerbyi*, three of *M. siligorensis*, two of *M. alticraniatus*, and two of *M. phanluongi*. The drawings of the baculum *M. alticraniatus* and *M. phanluongi* were taken from the literature (Borisenko et al. 2008; Kruskop 2013a).

### ﻿Molecular studies

All samples in which the cytochrome b (*Cytb*) and 12S ribosomal DNA (12S rDNA) genes were amplified and were obtained from specimens deposited at the Museum of Natural History of Northeast Normal University, Jilin Province, China. Total genomic DNA was extracted from the muscle tissues using a UNIQ-10 column animal genomic DNA isolation kit (Sangon, Shanghai, China).

The *Cytb* gene of all samples was amplified using the universal primers L14724/H15915 (Kocher et al. 1989; Irwin et al. 1991), whereas the 12S rDNA gene was amplified with the primer pair 12c/12g (Springer et al. 1995). Each PCR mixture contained 50 ng of genomic DNA, 10 mM Tris-HCl, 50 mM KCl, 1.5 mM MgCl_2_, 200 μM of each dNTP, 0.5 μM of each primer, and 2.5 U of Taq DNA polymerase (TaKaRa, Dalian, China) in a total volume of 25 μL. The PCRs were carried out in a thermocycler for 5 min at 94 °C, followed by 35 cycles of 45 s at 94 °C, 45 s at 44 °C, and 90 s at 72 °C, followed by one final extension at 72 °C for 5 min.

The PCR products were purified using an EZ-10 Spin column DNA Gel extraction kit (BBI, Shanghai, China) and then sequenced using an ABI PRISM 3730 sequencer (Applied Biosystems, Foster City, USA). The GenBank accession numbers are given in Table 1.

**Table 1.** Origin of the samples analyzed for Cytb and 12S rDNA.

Species	Cytb	12S rDNA	Reference/voucher (Cytb; 12S rDNA)
*M. badius*	GD-08-38	GD-08-38	KF894921; KF894928
	GX-07-10	GX-07-10	KF894922; KF894929
	MW054891.1		Ruedi et al. 2021
*M. kalkoae* sp. nov.	HUN-08c-16	GZ-07-74	KF894923; KF894930
	CQ-08c-16		KF894920
*M. sowerbyi*	AH-08-9	AH-08-9	KF894919; KF894927
	ZJ-08-47	JS-08-31	KF894926; KF894931
	JS-08-31	JX-09-89	KF894924; KF894932
	JS-08-37		KF894925
*M. alticraniatus*	OR096759.1	AY495508.1	Liu et al. 2023; Hoofer et al. 2003
		FJ755898.1	Borisenko et al. 2008
*M. davidii*	EF570884.1		hn1161
*M. muricola*	AJ841957.1	FJ755896.1	Stadelmann et al. 2004
			Borisenko et al. 2008
*M. capaccinii*	AF376845.1	AY495494.1	Ruedi et al. 2001
			Hoofer and Van Den Bussche 2003
*M. ikonnikovi*	AY665162.1		Tsytsulina et al. 2012
*M. altarium*	FJ215677.1		Zhang et al. 2009
*M. mystacinus*	AF376861.1		Ruedi and Mayer 2001
*M. brandtii*	AF376844.1		Ruedi and Mayer 2001
*M. longipes*	EF555231.1		Zhang et al. 2009
*M. petax*	EF555237.1		(Tan and Feng, unpublished)
*M. frater*	FJ215682.1		Zhang et al. 2009
*M. phanluongi*		FJ755897.1	Borisenko et al. 2008
*M. annamiticus*		FJ755901.1	Borisenko et al. 2008
*M. csorbai*		FJ755892.1	Borisenko et al. 2008
*M. alcathoe*	AJ841955.1		Stadelmann et al. 2004
*Kerivoula hardwickii*	GU585657	FJ755904.1	Khan et al. 2010
			Borisenko et al. 2008
*Kerivoula titania*	JN112246	FJ755902.1	Wu et al. 2012
			Borisenko et al. 2008

In order to investigate the phylogenetic position of these bats, the available *Cytb* sequences and 12S rDNA genes of the other bats were obtained from GenBank (Table 1). Molecular analyses of the *Cytb* and 12S rDNA data were performed based on the maximum likelihood (ML) criteria, using the MEGA11 molecular genetic analysis software (Tamura et al. 2021). The maximum likelihood search used the maximum composite likelihood method and pairwise deletion of the missing data. The tree was rooted with a composite outgroup containing *Kerivoula titania* and *K. hardwickii* (basal Vespertilionids, which were also used as an outgroup in Borisenko et al. 2008). The reliability of the nodes of ML phylogenetic trees was assessed by performing 1000 non-parametric bootstraps (Felsenstein 1985), and nodes with over 70% bootstrap support were considered strongly supported (Hillis and Bull 1993).

## ﻿Results

### ﻿Molecular results

**Figure 2.**
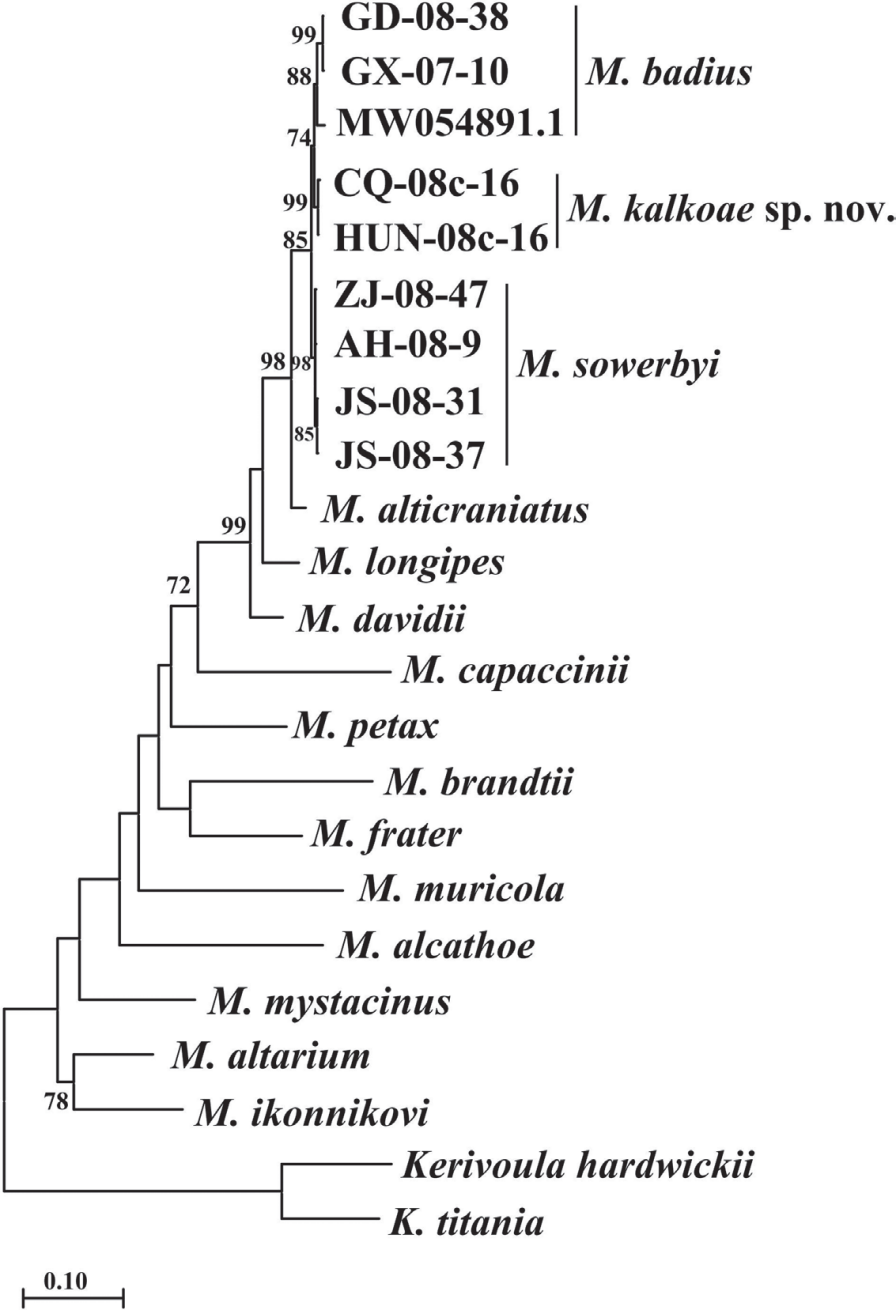
Maximum likelihood tree constructed using complete cytochrome b gene sequences. The nodes were considered supported if the indicated bootstrap exceeded 70%.

**Figure 3.**
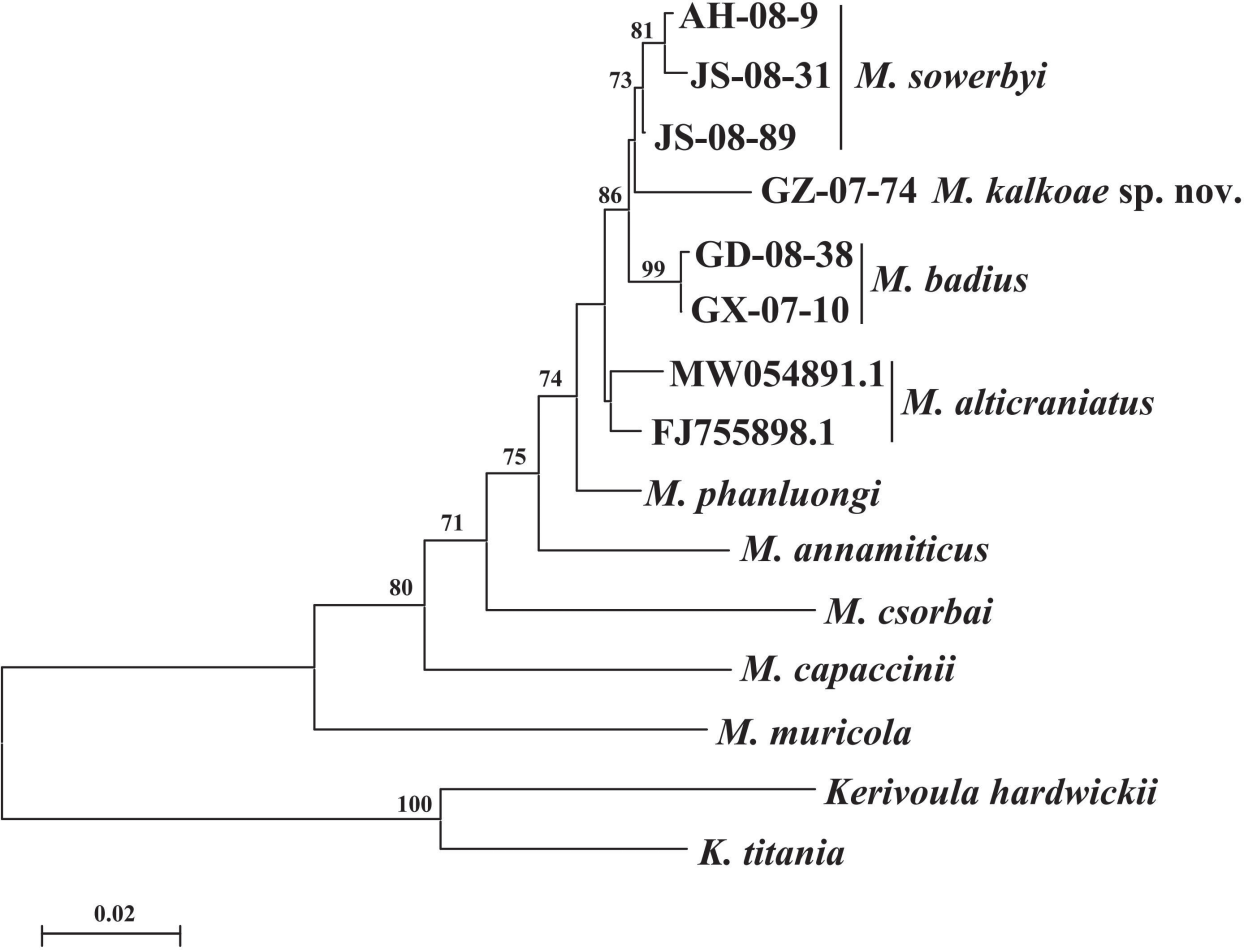
Maximum likelihood tree constructed from the 12S rDNA gene sequences. The nodes were considered supported if the indicated bootstrap exceeded 70%.

The genetic differences between the specimens of *Myotis kalkoae* sp. nov., *M. sowerbyi*, and *M. badius* were minimal (Suppl. materials 2, 3). The divergence in the *Cytb* gene between each species pair did not exceed 2.0% (Fig. 2). Nevertheless, each of the three forms generated a monophyletic clade with high bootstrap support. The 12S rDNA gene was significantly different between at least *M. kalkoae* sp. nov. and *M. badius* at ~2.2% (Fig. 3). All three Chinese species formed a highly supported monophyletic clade, with *M. alticraniatus* as a sister group.

The three Chinese forms, as well as other studied members of the ‘*siligorensis*’ species group, possess certain qualitative morphological differences in tragus and baculum shapes. These characteristics are described in the Systematics section below. Different bacular shapes were associated with particular genetic lineages. The distribution of bats with different bacular morphologies essentially corresponds to the distribution of the different genetic races of ‘*M. davidii*’ and the accepted subspecies of *M. siligorensis*. Animals from one of the genetically differentiated forms assigned previously to ‘*M. davidii*’ from the Chinese provinces of Yunnan were recently described as a separate species, *Myotis badius* Tiunov, Kruskop & Feng, 2011 (Tiunov et al. 2011).

**Figure 4.**
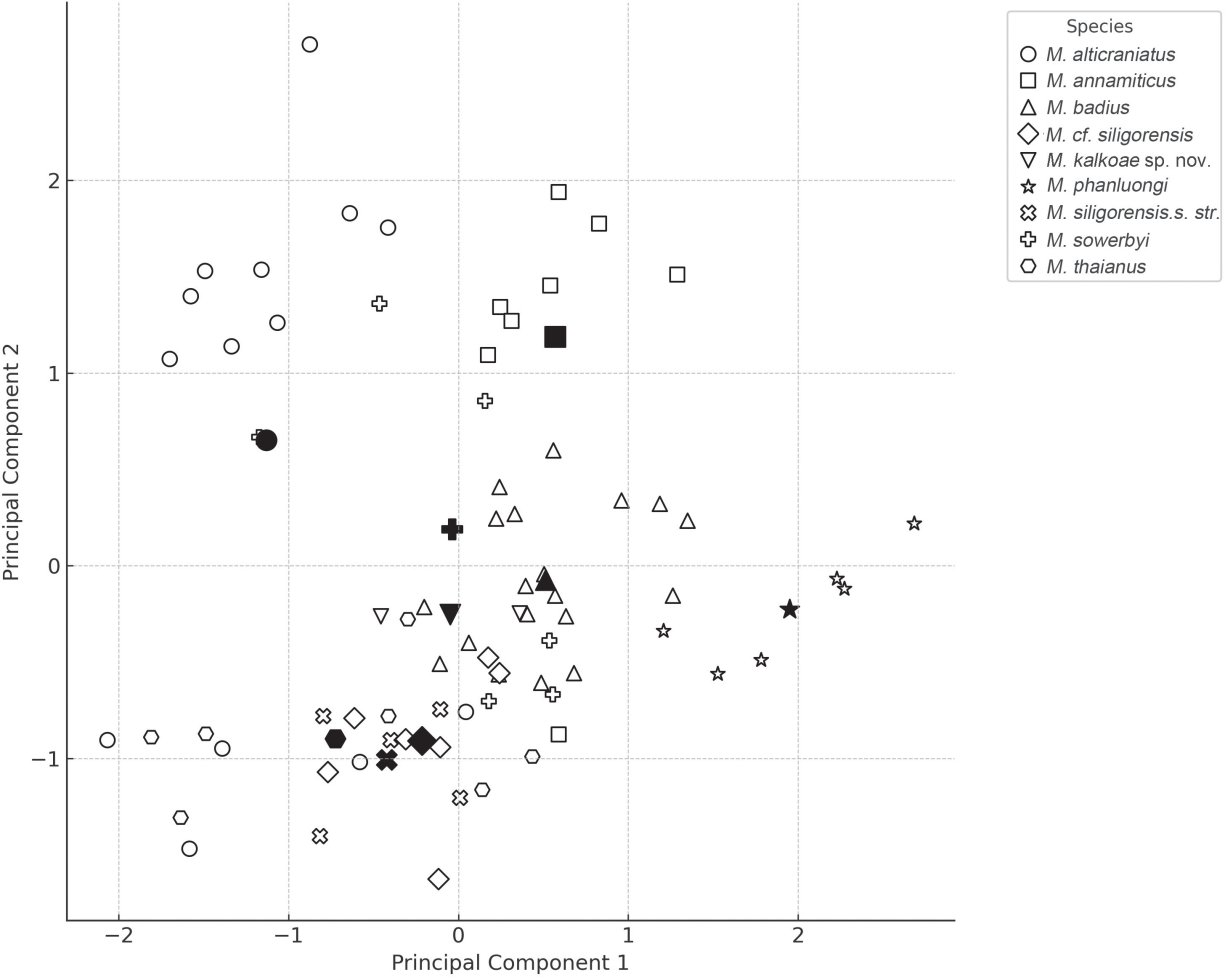
Bivariate scatter plot for the first and second principal components, which was generated through calculations related to the 17 cranial and dental measurements of the 74 specimens of Asiatic mouse-eared bats from the *M. siligorensis* species group. Factor loadings and eigenvalues are presented in Table 2. The filled symbols indicate centroids.

Moreover, the degree of skull variation between the forms is low. A PC analysis, conducted with different datasets to include or exclude the partly damaged samples, clearly divided at least a portion of the studied taxa. A bivariate scatter plot, shown in Fig. 4, was generated based on the two ‘first principal components’, which were calculated from the set of all seventeen craniodental measurements taken for the 74 samples of only the nyctalodont *Myotis* species. These two principal components cumulatively accounted for ~70.8% of the total variance (Table 2). PC I was positively correlated with the overall skull size and length of the upper and lower tooth rows; PC II was positively correlated with the length and width of the rostrum. *Myotis annamiticus* and *M. phanluongi* were plotted, and no overlap with other species was noted; for *M. badius*, this overlap was minimal. Moreover, in this particular analysis, *M. alticraniatus* and *M. sowerbyi* could not be fully separated from *M. siligorensis*. In another analysis with a partially reduced dataset, *M. alticraniatus* was well separated from at least *M. sowerbyi* based on the first and third PCs (the third one exhibited a high positive correlation with the width and height of the braincase). Only the animals from North Vietnam and adjacent areas of China were included in the analysis as *M. alticraniatus* individuals, while the other samples from Central Vietnam were included as ‘M. cf. siligorensis’ because their species affiliation was unclear and could not be determined from the geographical data. *Myotis kalkoae* sp. nov. tended to group with *M. badius*.

**Table 2.** Factor loadings and eigenvalues for the first four factors of the principal component analysis (refer to Materials and methods section for measurement explanations).

Variables	Factor 1	Factor 2	Factor 3	Factor 4	Factor 5
CBL	0.880909	0.088008	0.349225	0.026806	0.202124
CCL	0.87299	0.098803	0.382613	-0.01781	0.118612
W	0.488008	0.069929	0.667296	0.237199	0.384669
BCW	0.376094	0.155454	0.826189	0.039022	0.121379
BCH	0.432414	0.210102	0.755504	0.06296	0.216788
IOW	0.22236	0.143539	0.871806	0.157972	-0.03859
WR	0.121774	0.83235	0.408693	0.175527	0.033984
LR	0.0085	0.926508	0.063238	-0.2437	-0.10251
CM3	0.938647	0.111915	0.230636	0.000502	0.115725
C	0.408108	0.05006	0.333495	0.662105	0.21992
PD	0.348308	0.410706	0.002371	-0.74466	-0.12945
P4M3	0.844342	-0.04997	0.234004	0.314603	0.251428
CC	0.619659	0.080742	0.459453	0.496723	-0.03456
M3M3	0.524295	0.250134	0.1043	0.466663	0.408812
LMD	0.835609	0.099881	0.343438	0.091036	0.219735
HMD	0.396684	-0.18501	0.177427	0.230937	0.806367
MCM3	0.915714	0.025498	0.246577	0.006494	0.124716
Eigenvalue	9.734514	2.299991	1.601145	0.852859	0.525395
% total variance	57.26184	13.52936	9.418501	5.016816	3.090561
Cumulative eigenvalue	9.734514	12.0345	13.63565	14.48851	15.0139
Cumulative % of variance	57.26184	70.79121	80.20971	85.22652	88.31708

However, only two samples of *M. kalkoae* sp. nov. were included in the PC analysis, and this data may, therefore, be inadequate. In the DF analysis, based on the same dataset, no significant difference was found between *M. alticraniatus* and *M. siligorensis* (Table 3). The specimens of *M. kalkoae* sp. nov. were not distant from those of *M. sowerbyi* and *M. siligorensis thaianus*, but they were separated from the centroids of any group by greater distances than some groups were from each other.

**Table 3.** Distances between the groups, which were calculated for the set of 17 craniodental measurements taken from 74 *Myotis* samples, and the squared Mahalanobis distances from each learning sample for the two samples of *M. kalkoae* sp. nov. The squared Mahalanobis distances between the centroids of the ‘learning’ samples are shown above the diagonal. The p-distance values are shown below the diagonal. The ‘learning’ samples were established based on the genetic (when possible) and geographic data.

Species	М. badius	М. phanluongi	М. annamiticus	М. alticraniatus	М. sowerbyi	М. siligorensis	М. thaianus
*М. badius*		50.0468	80.2135	89.5715	48.7081	87.6536	62.8694
*М. phanluongi*	0.0000		48.9781	62.9411	40.3858	57.3186	45.9610
*М. annamiticus*	0.0000	0.0000		49.6433	34.2495	70.0562	75.5524
*М. alticraniatus*	0.0000	0.0000	0.0000		35.1336	10.6846	20.5998
*М. sowerbyi*	0.0000	0.0000	0.0000	0.0000		43.8002	22.3079
*М. siligorensis*	0.0000	0.0000	0.0000	0.0037	0.0000		12.3070
*М. thaianus*	0.0000	0.0000	0.0000	0.0001	0.0009	0.0317	
*М. kalkoae* sp. nov. HUN-08c-24	30.2260	49.7453	73.6164	63.4133	23.2860	65.5828	33.0132
*М. kalkoae* sp. nov. CQ-08c-16	62.9663	96.8092	123.1727	79.6705	43.2969	79.0009	41.6888

Despite the craniometric results, the presence of qualitative morphological features in combination with the level of genetic difference allowed us to suggest a specific rank for the unnamed form from China.

### ﻿Taxonomic account

#### ﻿ Myotis kalkoae

Taxon classificationAnimaliaChiropteraVespertilionidae

Tiunov, Jiang & Liu sp. nov.

5751E993-8969-53CA-A1C4-9329D338E802

https://zoobank.org/BD6560B6-4AF2-4F4D-8728-289768EFEFA3

Figs 5A, 6A, 7

##### Type material.

***Holotype*.** • IBSS CQ-08c-16, adult male, body in 75% alcohol, skull and baculum extracted, collected by Liu Sen in July 2008 from Shenxian Cave, near Jiangkou village, Wulong Town, Chongqing Province, China (29°16'451"N, 107°50'495"E). ***Paratypes*.** • IBSS HUN-08c-24, adult male, body in alcohol, skull extracted, collected by Sen Liu in July 2008 from Tangle Cave, near Zhaiyang village, Jishou Town, Hunan Province, China (28°17'927"N, 109°39'364"E); • NNU GZ-07-74, adult male, body in alcohol, skull extracted, collected by Sen Liu in July 2008 from Shizilu Cave at Dongnuang Village (28°18'539"N, 106°12'367"E).

##### Diagnosis.

A *Myotis* species of small size was established as belonging to the *siligorensis* species group, with the following characteristics: forearm length = 34.9, 35.3 mm (*n* = 2), condylobasal length of skull = 11.9, 12.1 mm (*n* = 2). The margin of the plagiopatagium was attached to the metatarsus of the first toe. The foot, including the claw, accounted for 50% (49, 51%, *n* = 2) of the tibia. The frontal portion of the skull was distinctly elevated above the low rostrum, as in *M. siligorensis*, *M. badius*, *M. csorbai*, and *M. annamiticus*. Both small upper premolars (P^2^ and P^3^) were present in the tooth row and clearly visible from a lateral view. The lower molars were of the seminyctalodon type, as in *M. siligorensis*, *M. badius*, *M. phanluongi*, and *M. annamiticus*. This new form could be clearly distinguished from all the latter species by the shape of the baculum. The measurements of the holotype (in mm) were as follows: body length – 35, tail length – 28, length of the free tail tip – 0.5, ear length – 11.9, tragus length – 5.5, tibia length – 12.4, foot length with claws – 7.3, forearm length – 34.9, length of the first digit (without claws/with claws) – 3.6/5.0, Mc2 – 28.2, Mc3 – 29.8, Ph3.1 – 9.3, Ph3.2 – 8.2, Ph3.3 – 6.4, Mc4 – 29.1, Ph4.1 – 9.4, Ph4.2 – 8.3, Mc5 – 29.1, Ph5.1 – 8.4, Ph5.2 – 7.8; CBL – 11.9, CCL – 11.1, W – 6.8, BCW – 6.4, BCH – 5.6, IOW – 3.2, ZW – 8.0, WR – 4.1, LR – 2.95, CM3 – 4.5, PD – 0.5, P4M3 – 3.45, WM3 – 1.25, LM3 – 0.6, CC – 3.3, M3M3 – 5.2, LMD – 9.3, HMD – 2.5, MCM3 – 4.9.

##### Description.

**Figure 5.**
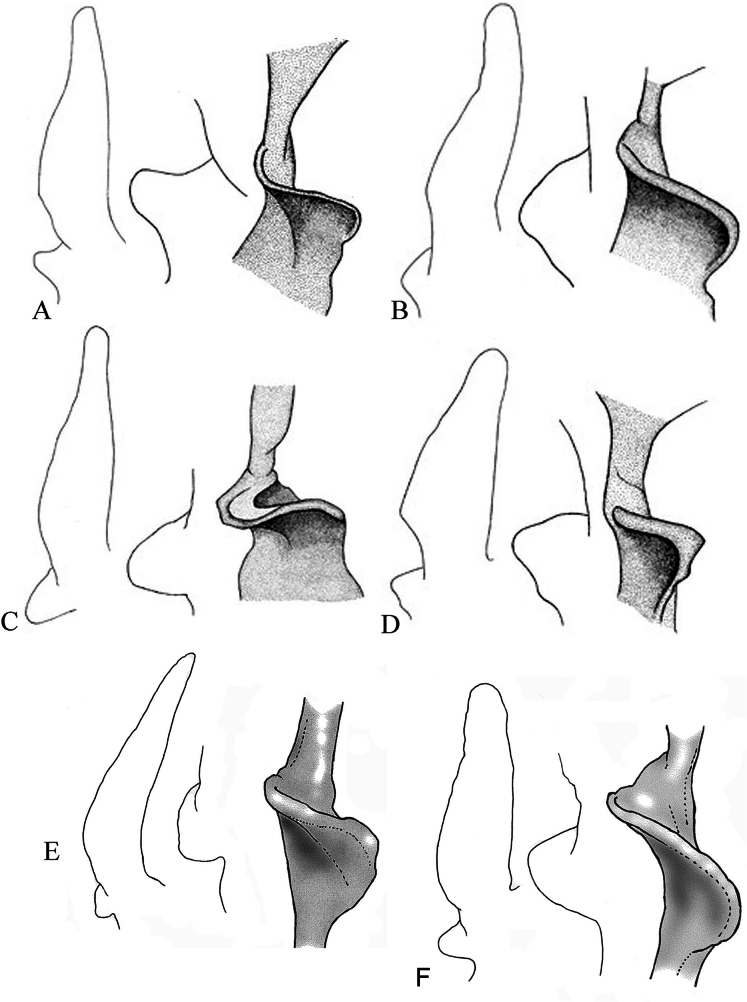
Shape of the tragus and lobe at the base of the tragus: A. *Myotis kalkoae* sp. nov. (tragus, lobe at the base of the tragus (anterior view, view from above and lateral view)); B. *Myotis sowerbyi* (tragus, lobe at the base of the tragus (anterior view, view from above and lateral view)); C. *Myotis badius* (tragus, lobe at the base of the tragus (anterior view, view from above and lateral view)); D. *Myotis siligorensis* (tragus, lobe at the base of the tragus (anterior view, view from above, and lateral view)); E. *Myotis alticraniatus* (tragus, lobe at the base of the tragus (anterior view, view from above, and lateral view)); F. *M. phanluongi* (tragus, lobe at the base of the tragus (anterior view, view from above, and lateral view)).

The hairs of the dorsal pelage were brown with a grayish tinge. The fur was uniformly paler on the ventral surface. Each ear was comparatively short, not extending, when laid forward, to the tip of the nose. The tragus was relatively short, a little less than 1/2 as high as the conch. The tragus’s anterior border was straight with a rounded tip, and its widest portion was in the lower 2/3. The lobe at the base of the tragus was thin, wavy, curved, and perpendicular to the tragus. The outer angle exhibited a mastoid form and points upward (Fig. 5A). The wing membrane was broad, and the fifth finger was relatively long, ~84% of the third finger in length. The calcar was ~35% (32.2, 43.0%, *n* = 2) the length of the posterior border of the interfemoral membrane when measured from the foot to the tip of the tail; it was without a keel or terminal lobe. The tail was relatively long, at 82% of the head and body length; ~0.45 mm of its tip was free from the membrane. The ventral surface of the interfemoral membrane was sparsely haired, and the hairs were ~0.7–0.8 mm in length.

**Figure 6.**
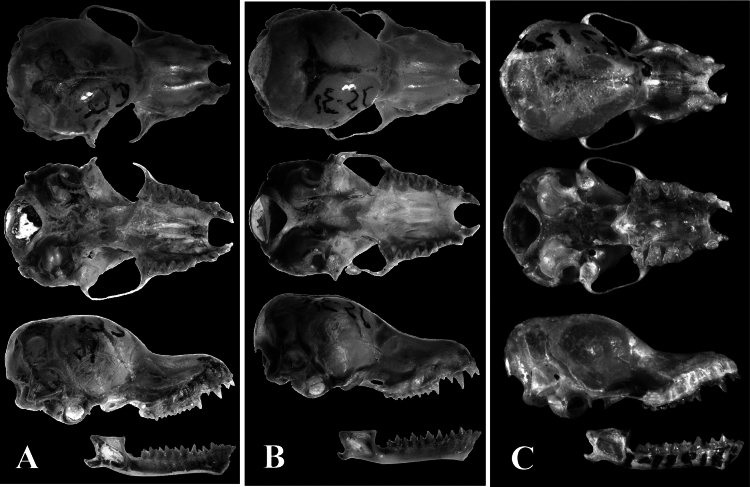
A. Dorsal, ventral, and lateral views of the cranium and mandible of *Myotis kalkoae* sp. nov. (holotype); B. Dorsal, ventral, and lateral views of the cranium and mandible of *Myotis sowerbyi*; C. Dorsal, ventral, and lateral views of the cranium and mandible of *M. alticraniatus*.

The frontal portion of the skull was distinctly elevated above the low rostrum, as in *M. csorbai*, *M. badius*, and *M. siligorensis*. The height of the brain sample was ca. 78.9% (75.4, 82.35%, *n* = 2) of the skull width. The upper surface of the rostrum contained a visible groove in the middle (Fig. 6A). The interorbital constriction was remarkably narrow; the interorbital width was ~46% of the skull width. The posterior border of the naris extends to the posterior margin of the upper canine. The sagittal crest was scarcely evident, and the lambdoid crests were visible laterally but were lacking in the central portion. The outer upper incisor (I^3^) possessed three or four cusps and was equal to or slightly greater than the internal incisor (I^2^). The upper canine was short; it scarcely exceeded the posterior premolar (P^4^) in height and occupied a smaller crown area. The anterior premolar (P^2^) was in contact with the canine and < 1/2 of its height. The middle premolar (P^3^) was small, < 2/3 of P^2^ in height and ~1/2 the crown area. Both small upper premolars were situated in the toothrow and were visible when the skull was viewed laterally. The posterior premolar (P^4^) was low, with an antero-internal cusp. The upper molars possessed well-developed protoconules. In the lower dentition, the first (I_1_) and second lower incisors (I_2_) were three- or four-lobed. The canine was small, and its height was less than that of the posterior lower premolar (P_4_). The first lower premolar (P_2_) was ~2/3 the height of the canine. The second lower premolar (P_3_) was ~1/2 the height of P_2_ and was located within the toothrow. The lower first molar was of the nyctalodont type (the postcristid was connected to the hypoconulid). The second molar was of a seminyctalodont dental type (the postcristid connects to both the entoconid and the hypoconulid). The third molar was a typical myotodont.

**Figure 7.**
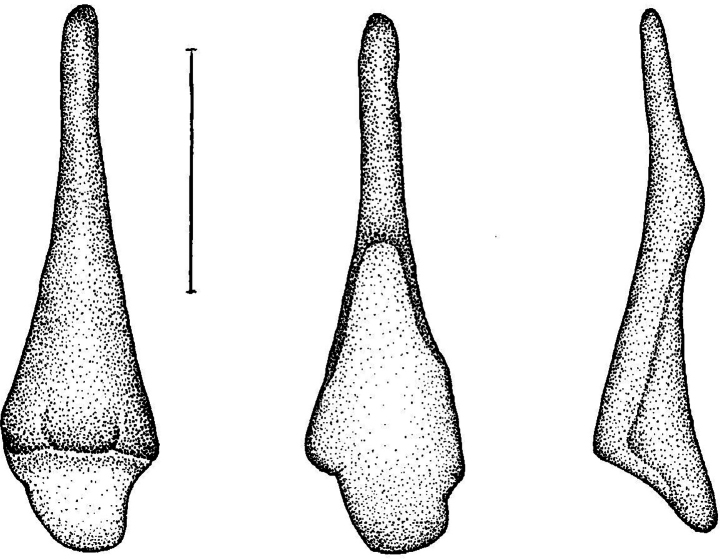
Baculum of *Myotis kalkoae* sp. nov., dorsal, ventral, and lateral views. Scale bar: 0.2 mm.

Baculum. The baculum of *M. kalkoae* sp. nov. was essentially similar to that of the other species within this group. The feature was a small bonelet that was broadly rounded proximally and narrowed distally. Viewed laterally, it was recurved in shape (Fig. 7). A reduced urethral groove was present proximally on the ventral surface; it appeared as a triangularly shaped, poorly articulated depression. The length was ~0.5 mm; the greatest width was ~0.15 mm (*n* = 2 adults).

##### Etymology.

The new species is named in honor of Dr. Elisabeth Kalko (1962–2011), head of the Institute of Experimental Ecology (University of Ulm, Germany), who, in the early 2000s, was one of the most prominent experts in the field of bat community ecology.

## ﻿Discussion

Individuals of the new species were compared with the following members of the *M. siligorensis* group: *M. alticraniatus*, *M. badius*, *M. phanluongi*, *M. s. siligorensis*, *M. sowerbyi*, and *M. s. thaianus*.

### ﻿*Myotis sowerbyi* Howell, 1926

The form sowerbyi was described based on a single female specimen (Howell 1926; specimen no. 238869 in the collection of the National Museum of Natural History, Washington) and subsequently treated as a Chinese subspecies of *M. siligorensis* (Corbet 1978; Koopman 1994; Simmons 2005). Unfortunately, the holotype could not be directly investigated by the authors: the available photographs of this sample corresponded well to the individuals at our disposal in terms of overall and skull shapes and proportions. However, there is some controversy in the opinions about the molar type of the *M. sowerbyi* holotype, which should be clarified in further studies.

Several males were captured in four Chinese provinces (Jiangxi, Anhui, Zhejiang, and Jiangsu Provinces), both westward and northward from the species type locality (Fujian Province), and their measurements are as follows (in mm): forearm length 33.1–35.29 mm (mean = 33.98; *n* = 8), body length 37.11–41.2 (39.3; *n* = 3), tail length 31.8–37.38 (35.0; *n* = 8), calcar length 10.42–15.00 (12.36; *n* = 9), length of free tail tip 0.5, ear length 11.0–12.66 (11.86; *n* = 7), tragus length 5.88–6.74 (6.16; *n* = 7), tibia length 14.74–16.35 (15.59; *n* = 8), foot length with claws 7.2–8.7 (7.77; *n* = 3), length of the first digit (without claws and with claws) 4.3–4.6/5.1–6.4 (4.43/5.93; *n* = 3), Mc2 – 27.1–29.7 (28.5; *n* = 3), Mc3 – 28.52–31.40 (30.27; *n* = 8), Ph3.1 – 9.67–11.2 (10.44; *n* = 6), Ph3.2 – 8.29–9.35 (8.70; *n* = 6), Ph3.3 – 5.00–6.55 (5.99), Mc4 – 28.38–30.49 (29.71; *n* = 6), Ph4.1 – 8.08–9.84 (9.13; *n* = 6), Ph4.2 – 8.20–8.68 (8.36; *n* = 6), Mc5 – 27.83–30.07 (29.37; *n* = 6), Ph5.1 – 8.04–9.32 (8.52; *n* = 6), Ph5.2 – 7.34–8.01 (7.72; *n* = 6); and CBL – 12.1–12.3 (12.2; *n* = 3), CCL – 11.25–11.4 (11.33; *n* = 3), W – 6.5–6.9 (6.73; *n* = 3), BCW – 6.35–6.5 (6.45; *n* = 3), BCH – 5.6, IOW – 3.2–3.5 (3.3; *n* = 3), ZW – 7.3, WR – 3.75–4.0 (3.88; *n* = 3), LR – 2.8–3.0 (3.9; *n* = 3), CM3 – 4.7–4.75 (4.73; *n* = 3), PD – 0.75 (*n* = 3), P4M3 – 3.30–3.35 (3.32; *n* = 3), WM3 – 1.1–1.2 (1.13; *n* = 3), LM3 – 0.6–0.65 (0.62; *n* = 3), CC – 3.25–3.4 (3.32; *n* = 3), M3M3 – 5.2 (4.95–5.05; *n* = 3), LMD – 9.3–9.6 (9.47; *n* = 3), HMD – 2.25–2.5 (2.42; *n* = 3), MCM3 – 5.0–5.05 (5.03; *n* = 3).

This bat was, based on the external, cranial, and dental features, most similar to *Myotis kalkoae* sp. nov. The inner edge of the tragus was curved, with the tip slightly bent outward. The maximum width of the tragus was in the lower third toward the base. The lobe at the base of the tragus was thicker than that of *M. kalkoae* sp. nov. The lobe’s exterior angle was triangular in shape, evenly rounded, and horizontally distributed (Fig. 5B).

The margin of the plagiopatagium was attached to the metatarsus of the first toe. The length of the foot, including the claw, was ~50% (46%–54%, *n* = 3) of the length of the tibia. The frontal portion of the skull was distinctly elevated above the low rostrum (Fig. 6B). The height of the brain sample was ~76.8% (72.5%–80.0%, *n* = 3) of the skull width. The interorbital constriction was remarkably narrow; the interorbital width was ~49% of the skull width. The posterior border of the naris extended to a level comparable to the middle of the upper canine. The sagittal crest was scarcely evident, and the lambdoid crest was visible laterally but lacking in the central portion. The outer upper incisor (I^3^) contained three cusps and was equal to or slightly greater than the internal incisor (I^2^). The upper canine was short; it scarcely exceeded the posterior premolar (P^4^) in height and occupied a smaller crown area. The anterior premolar (P^2^) was in contact with the canine and was ~1/2 its height. Both the small upper premolars (P^2^ and P^3^) were situated in the toothrow and were clearly visible in a lateral view. The lower first molar was a nyctalodont, the second molar was of a seminyctalodont dental type, the third molar was a myotodont.

**Figure 8.**
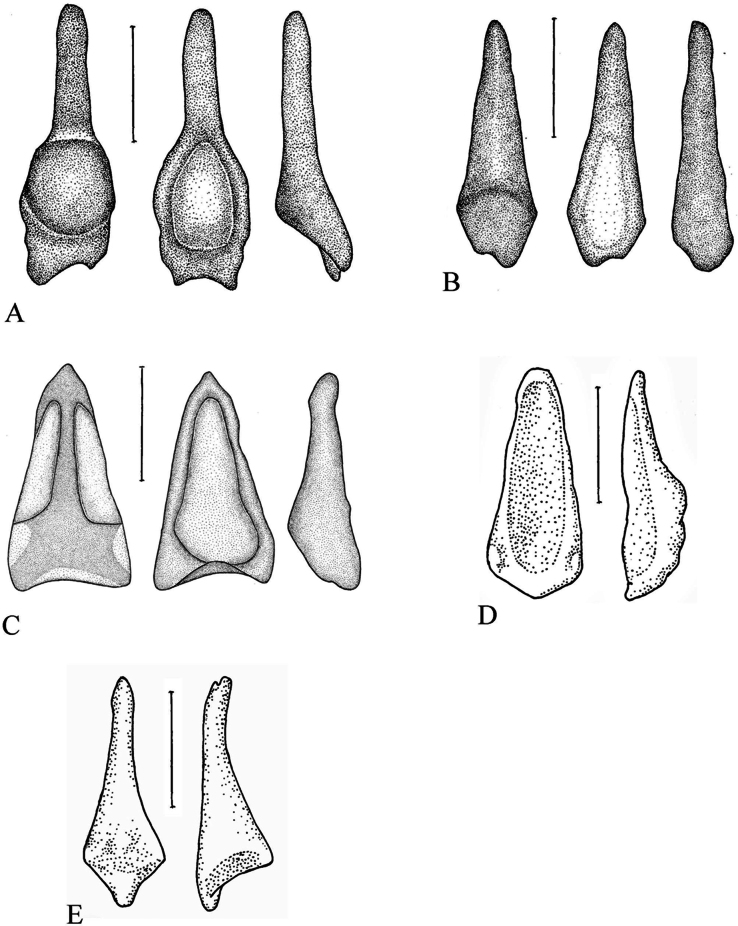
Bacula of the selected *Myotis siligorensis* species group. A. *Myotis sowerbyi* (dorsal, ventral, and lateral views); B. *Myotis badius* (dorsal, ventral, and lateral); C. *Myotis siligorensis* (dorsal, ventral, and lateral); D. *Myotis alticraniatus* (ventral and lateral); E. *Myotis phanluongi* (dorsal and lateral). Scale bars: 0.2 mm.

Baculum. The penial bone was small and spoon-like in general shape. A reduced urethral groove, in the form of a deep oval-shaped depression, was present on the proximal half of the ventral surface. On the proximal part, a wide oval incision with protruding asymmetrical spikes was observed (Fig. 8A). The greatest length was ~0.5 mm; the greatest width was ~ 0.18 mm (*n* = 8 adults).

### ﻿*Myotis alticraniatus* Osgood, 1932

This bat was described based on a sample from Muong Muon in the western mountainous part of Northern Vietnam. Until recently, this form was treated as a subspecies of *M. siligorensis* (Koopman 1994; Kruskop 2013b). This individual is a very small bat with a forearm length of 30–35 mm (33.13, *n* = 14) and a CCL of 10.0–10.84 mm; externally, it is similar to *M. sowerbyi* and *M. siligorensis*, although with a proportionally smaller skull.

The inner edge of the tragus was curved, with the tip slightly bent forward. The maximum width of the tragus was in the lower third near the base. The upper part of the tragus was relatively narrow and pointed in shape. The lobe at the base was thicker than those of *M. kalkoae* sp. nov. and of *M. sowerbyi*. The lobe’s external angle was a bluntly rounded shape, with the apex directed downwards (Fig. 5E).

The margin of the plagiopatagium was attached to the metatarsus of the first toe. The length of the foot with the claw was ~50% (42%–53%, *n* = 10) of the tibia length. The frontal part of the skull was distinctly elevated above the rostrum (Fig. 6C). The brain sample was inflated, and its height was ~81.3% (78.4%–84.0%, *n* = 13) of the skull width. The interorbital constriction was narrow; the interorbital width was ~47%–51% of the skull width. The posterior border of the nasal emargination extended to a level comparable to the middle of the upper canine. Sagittal and occipital crests were almost absent, and the lambdoid crests were reduced but visible laterally. The outer upper incisor (I^3^) was equal in size to or slightly longer than the internal incisor (I^2^). The upper canine was small, only slightly exceeded or was equal in height, and it was smaller in crown area than the corresponding larger premolar (P^4^). Both small upper premolars (P^2^ and P^3^) were essentially in the toothrow and clearly visible from a lateral view. The first lower molar was a nyctalodont type, and the second was a nyctalodont or seminyctalodont.

Baculum. The penial bone was very small, ~0.35 mm in length. The structure was simple in shape and proportionally wider than that in *M. sowerbyi* and *M. kalkoae* sp. nov. The baculum was wider basally, evenly narrowing to the distal end without any abrupt constrictions. The urethral groove was reduced but was present as a depression on the lower surface of the bone and projects forward, almost to its tip (Fig. 8D).

### ﻿Remarks

Traditionally, all *siligorensis*-like *Myotis* individuals from Vietnam and adjacent parts of China and Laos have been referred to as *M. s. alticraniatus* (Kruskop 2013b). However, the genetic diversity of the COI gene in Indochinese and southern Chinese *M. siligorensis* sensu lato (Francis et al. 2010) exceeded the intraspecific level of variation common for other *Myotis* species. This suggests the existence of more than one species within that region, with an uncertain taxonomic position of animals from central Indochina. Thus, it is suggested to tentatively restrict the distribution of *M. alticraniatus* sensu stricto to the northern provinces of Vietnam and Laos and the adjacent territories of China (Fig. 1). Additional studies with different gene markers are, however, needed to conclude this topic.

The new species was slightly longer than *M. siligorensis*: the forearm length was 34.9 and 35.3, the condylobasal length was 11.9 and 12.1 vs. 29.1–29.5 and 10.2–10.8 mm in *M. s. thaianus* (data of this study) and 30.8 and 11.2 mm in *M. s. siligorensis* (Bates and Harrison 1997), respectively. *Myotis kalkoae* sp. nov. clearly differed from *M. siligorensis* in terms of baculum shape. The baculum in *M. siligorensis* was wedge-shaped with small lateral ‘wings’ (Fig. 8C). The structure was shorter but wider at the base than that of *M. kalkoae* sp. nov. (Fig. 7). The maximum baculum length was 0.45 mm; the maximum baculum width was 0.225 mm. The maximum width/length ratio of the baculum of *M. siligorensis* was 0.5, that of *M. alticraniatus* was 0.4, of *M. sowerbyi* was 0.36, and of *M. kalkoae* sp. nov. was 0.32.

*Myotis kalkoae* sp. nov. clearly differs in the tragus shape from those of *M. s. siligorensis* and *M. s. thaianus* (these two species have the same tragus shape). In contrast to *M. kalkoae* sp. nov., the tragus of *M. siligorensis* was lancet-shaped and wide at the base, tapering evenly to the top (Fig. 5D). *Myotis kalkoae* sp. nov. differs from *M. siligorensis* in terms of the maximum width/length ratio of the tragus. In *M. kalkoae* sp. nov., the ratio was 0.29, whereas in *M. siligorensis*, *M. badius*, and *M. sowerbyi*, the ratios were 0.38, 0.27, and 0.26, respectively. The lobe at the base of the tragus of *M. kalkoae* sp. nov. was thinner than that of *M. sowerbyi* and *M. badius*. The lobe’s exterior angle was directed upward, which is in contrast to *M. sowerbyi*, in which it was directed horizontally, and to *M. badius*, in which it was angled downward.

Most members of the *Myotis siligorensis* species group were very similar morphologically and could barely be distinguished based on traditional features such as skull proportions. Therefore, a taxonomic investigation is required to further research, with the evaluation of other morphological structures such as the baculum and shape of the tragi, and thorough attention to the peculiarities of the observed morphologies.

According to the results of a study by Ruedi (Ruedi et al. 2021), *M. badius* belongs to the subspecies *M. alticraniatus*. However, the structure of the baculum and tragus in the studied topotypes remains unknown to date. In addition, the distribution of *M. alticraniatus* in China remains unclear; therefore, this decision from Ruedi may be premature.

The results of the PC and DF analyses indicate very low skull variation within the *M. siligorensis* complex, which may explain why *M. alticraniatus*, *M. siligorensis*, and cf. *M. sowerbyi* were thought to be conspecific for so long. Most likely, a different set of measurements is required to discriminate the species. Unequal sample sizes could also have affected the results of the morphometric analyses. Finally, not all the samples were identified by bacular/ear morphology or by genetics and were assigned to a specific sample based on geographical distribution. Therefore, some misidentifications could have occurred if more than one species were present in the same region.

One of the main problems encountered in the clarification of the taxonomy of the *M. siligorensis* species group is the lack of genetic data, as few genes have been sequenced for the members of this group to date (Borisenko et al. 2008; Zhang et al. 2009; Francis et al. 2010; Ruedi et al. 2013), and only a few specimens have been sampled (Ruedi et al. 2021).

The identification of new cryptic forms expands the current knowledge of the taxonomic diversity of the *M. siligorensis* species group and highlights the intriguingly high species diversity within a morphologically uniform species complex. Most likely, the high diversity may be explained by the low mobility of the group members. The post-glacial periods of rapidly changing natural zones can easily lead to the appearance of isolated populations (e.g., those in separate mountainous ranges). The same scenario is most likely true for some other bats with the low mobility, such as *Murina* or *Rhinolophus* (Francis et al. 2010), for which many cryptic species have been recently identified (Csorba et al. 2011; Francis and Eger 2012; Patrick et al. 2013).

There are still a number of issues regarding the taxonomy of this group, which remain to be investigated in detail. The absence of suitable genetic material from a typical *M. siligorensis* from India and Myanmar, as well as that from *M. s. thaianus*, prevented the investigation of their genetic differences from each other and East Asian relatives. This difference, in light of the aforementioned results, can be considerable despite the morphological similarities. The relationships between the nyctalodont and myotodont members of the species group remain unclear. The authors are fairly confident that *M. laniger* is a member of this complex (see Ruedi et al. 2013), whereas the relationships between the morphologically similar species, such as *M. longipes* and *M. csorbai*, remain unknown. Therefore, further studies aimed at evaluating the phylogenetic history of the *M. siligorensis* complex require including novel material from all parts of the group distribution. Nuclear genetic markers should also be incorporated in future analyses because the similarities demonstrated only by the mitochondrial DNA may represent an artifact caused by a former gene flow, as reported for some Palaearctic bat species (Berthier et al. 2006; Artyushin et al. 2009, 2012).

## Supplementary Material

XML Treatment for Myotis kalkoae

## ﻿Additional information

### Use of AI

No use of AI was reported.

### Author ORCIDs

Mikhail Petrovich Tiunov https://orcid.org/0000-0002-4276-4266

Sen Liu https://orcid.org/0000-0003-2258-3676

Jiang Feng https://orcid.org/0000-0002-7503-1069

Pipat Soisook https://orcid.org/0000-0002-1404-3568

Tinglei Jiang https://orcid.org/0000-0003-3858-9458

### Data availability

In this study, all the gene sequences have been deposited in GenBank. The other data are available from the corresponding author (Mikhail Petrovich Tiunov: tiunov@biosoil.ru) upon reasonable request.

## Supplementary materials

Supplementary material 1 List of collection specimens used in morphological studies Data type docx https://binary.pensoft.net/file/1460313This dataset is made available under the Open Database License (http://opendatacommons.org/licenses/odbl/1.0/). The Open Database License (ODbL) is a license agreement intended to allow users to freely share, modify, and use this Dataset while maintaining this same freedom for others, provided that the original source and author(s) are credited.Mikhail Petrovich Tiunov, Sen Liu, Jiang Feng, Pipat Soisook, Tinglei Jiang

Supplementary material 2 Genetic distances based on Cytb sequences Data type xlsx https://binary.pensoft.net/file/1460314This dataset is made available under the Open Database License (http://opendatacommons.org/licenses/odbl/1.0/). The Open Database License (ODbL) is a license agreement intended to allow users to freely share, modify, and use this Dataset while maintaining this same freedom for others, provided that the original source and author(s) are credited.Mikhail Petrovich Tiunov, Sen Liu, Jiang Feng, Pipat Soisook, Tinglei Jiang

Supplementary material 3 Genetic distances based on 12S rDNA sequences Data type xlsx https://binary.pensoft.net/file/1460315This dataset is made available under the Open Database License (http://opendatacommons.org/licenses/odbl/1.0/). The Open Database License (ODbL) is a license agreement intended to allow users to freely share, modify, and use this Dataset while maintaining this same freedom for others, provided that the original source and author(s) are credited.Mikhail Petrovich Tiunov, Sen Liu, Jiang Feng, Pipat Soisook, Tinglei Jiang
