# Supplementary material for: ﻿A new species of Myotis from China with notes on the siligorensis species group (Chiroptera, Vespertilionidae)
Source: Zookeys. 2025 Nov 7;1258:333–56. doi: 10.3897/zookeys.1258.145290 (PMC12676132; doi:10.3897/zookeys.1258.145290)
Supplement: Supplementary material 1 — List of collection specimens used in morphological studies [file zookeys-1258-333_article-145290__-s001.docx]

**A new species of Myotis from China with notes on the 'siligorensis' species group (Chiroptera, Vespertilionidae)**

Mikhail Petrovich Tiunov^1^, Sen Liu^2^, Jiang Feng^3^, Pipat Soisook^4^, Tinglei Jiang^5^.

Supplementary file S1. List of collection specimens used in morphological studies.

*Myotis kalkoae* sp. nov. – China: Chongqing: IBSS CQ-08c-16 male (holotype); China, Hunan: IBSS HUN-08c-24 male, China, Guizhou: NNU GZ-07-74 male (paratypes).

*Myotis csorbai* Topal, 1997 – Central Nepal (including five paratypes of the species): ZMMU S-164475 female, ZMMU S-164476 female, ZMMU S-164478 female, ZMMU S-164481 female, ZMMU S-164483 female, ZMMU S-164484 female, ZMMU S-164485 male, ZMMU S-164487 male, ZMMU S-164490 male.

*Myotis annamiticus* Kruskop & Tsytsulina, 2000 – Central Vietnam (from the type series of the species): ZMMU S-167123 male, ZMMU S-167126 male, ZMMU S-167127 female, ZMMU S-167128 female, ZMMU S-167132 female, ZMMU S-167134 female, ZMMU S-167135 female.

*Myotis* cf. *annamiticus* – Laos: ROM 110661 male.

*Myotis phanluongi* Borisenko, Kruskop & Ivanova, 2008 – Vietnam Central Highlands (including a holotype and three paratypes): ZMMU S-175153 female, ZMMU S-175154 female, ZMMU S-175155 male, ZMMU S-175156 female, ZMMU S-182120 female, ZMMU S-191927 male, ZMMU S-191929 female.

*Myotis badius* Tiunov, Kruskop & Feng, 2011 – China, Yunnan: (from the type series of the species): IBSS u-06-88 male, IBSS u-06-8 male, IBSS u-08-80 male, IBSS u-06-13 female, IBSS u-06-14 female, IBSS u-06-15 female, IBSS u-06-16 female, IBSS u-06-20 female, IBSS u-06-22 female, IBSS u-06-28 female, IBSS u-06-34 female, IBSS u-06-35 female, IBSS u-06-13 female, ZMMU S-186520 male, ZMMU S-186521 female; China, Guangxi: NNU GX-07-10 male; China, Guizhou: NNU GZ-07-74 male.

*Myotis siligorensis siligorensis* Horsfield, 1855 – Nepal: BM(NH) 79.11.21.125 (holotype); India, Meghalaya: HZM. 11.36227 male; Myanmar: HZM10.36080 male; HZM8.35286 female, HZM13.29991 female.

*Myotis siligorensis thaianus* Shamel, 1942 – Southern Thailand, Satun: IBSS PSUZC-MM-06-61 male; Southern Thailand, Trang: IBSS PSUZC-MM-08-125 male; Southern Thailand, unspecified: SMF 88970 male; SMF 88971 male; SMF 88968 male; SMF 88963 male; SMF 88964 male; SMF 88966 male; MNH79.1417 male.

*Myotis alticraniatus* Osgood, 1932 – Northern Vietnam (Tuen Quang, Sa Pa and Muon Muon; including three females from the type series): ROM 107649 female, ROM 107652 female, ROM 107657 female, ROM 107677 female, ROM 112447 male, ROM 112456 male, ROM 112458 male, FMNH 32173 female, FMNH 32175 female, FMNH 32178 female, ZMMU S-186707 female, BM(NH) 1997.345 male; China, Guanxi: ROM 116118 female, ROM 116119 female.

*Myotis* cf. *alticraniatus* Osgood, 1932 – Vietnam Central Highlands: ZMMU S-175152 female, ZMMU S-175157 male, ZMMU S-175159 female; Central Vietnam: ZMMU S-167188 male.

*Myotis sowerbyi* Howell, 1926 – China, Jiangsu: IBSS JS-08-31 male, NNU JS-08-32 male; China, Anhui: IBSS AH-08-08 male, IBSS AH-08-10 male, IBSS AH-08-87 male; China, Zhejiang: NNU ZJ-08-47 male; China, Jiangxi: NNU JX-09-89. *Myotis laniger* (Peters, 1871) – Northern Vietnam: ROM 107655, ROM 107665 female, ROM 112492 male, ROM 111269 male, HNHM 93.57.1 female; India: HNHM 92.108.1 male, HNHM 92.108.2 male, HNHM 92.108.3 female; Southern China: IBSS u-06-75, IBSS u-06-106.

*Myotis* cf. *taiwanensis* Linde, 1908 – Taiwan: HNHM 2005.65.43.

*Myotis muricola* (Gray, 1846) – Southern Vietnam: ZMMU S-173413 male, ZMMU S-172616 male, ZMMU S-172626 male; Central Vietnam: ZMMU S-165048 female, ZMMU S-165055 male.

*Myotis* *davidii* Peters, 1869 – Central China: MNHN 1987-296 male (holotype), MNHN 1911-739 female, BM(NH) 9.1.1.1 male.
